# Inclusion of macroalgae in the diet – a comparative survey from Norway, Chile and China

**DOI:** 10.29219/fnr.v69.10856

**Published:** 2025-06-26

**Authors:** Franz Goecke, Inger Aakre, Lisse Angarita, Na Li, Xiaodong Li, María Cristina Escobar, Silvana Cisternas, Lianzhu Wang, Shaojun Pang, Åshild Ergon

**Affiliations:** 1 Department of Plant Sciences, Faculty of Biosciences, Norwegian University of Life Sciences, Ås, Norway; 2 Department of Seafood and Nutrition, Institute of Marine Research, Bergen, Norway; 3 Escuela de Nutrición y Dietética, Facultad de Medicina, Universidad Nacional Andres Bello, Sede Concepción, Concepción, Chile; 4 Yellow Sea Fisheries Research Institute, Chinese Academy of Fishery Sciences, Qingdao, P.R. China; 5 Institute of Oceanology, Chinese Academy of Sciences, Qingdao, P.R. China

**Keywords:** seaweed, nutrition, seafood, iodine, food risks, culinary traditions

## Abstract

**Background:**

Macroalgae have been an important dietary component in many parts of the world for centuries, especially in Eastern Asia. In recent years, a combination of factors has contributed to enhance the use of macroalgae as food in the global market. Since macroalgae as a commercially available food are new in many countries, only a handful of studies have investigated their use and consumption.

**Objective:**

In this tri-continental survey, we included three distant countries, each known for macroalgae producers with a long coast: Chile, China, and Norway. Our objective was to compare current uses of macroalgae as food, in a convenient sample dominated by male and female adult students.

**Design:**

A macroalgae-specific food frequency questionnaire with a 4-week recall period was used to assess intake frequencies, species, and product types among a convenient sample of Norwegian, Chinese, and Chilean students.

**Results:**

A total of 585 respondents who answered the survey considered macroalgae as appealing foods due to their flavor (23–67%) and nutritional benefits (49–90%). This study reported lack of awareness about potential food safety issues in this group. In the samples from Chile and China, tradition was important in terms of consumption of macroalgae, while food novelty seemed to be a major factor in the Norwegian group. However, all three countries consumed a similar number of species (17–19) and products containing macroalgae (17–18). Chinese respondents especially stood out for their frequency of consuming different products containing algae.

**Discussion and conclusion:**

A variety of species were found the diet in all the population groups, either in pure form or as an ingredient in a variety of products. Further research on macroalgae intake in Norway, including amounts consumed, would be useful to develop food regulations and, furthermore, recommendations that are commonly known to consumers.

## Popular scientific summary

- A survey conducted among Norwegian, Chinese and Chilean students (n=585), found that seaweed was widely consumed in this sample, with China showing highest frequency.
- Consumers value seaweed for taste and health benefits; tradition drives use in Asia and Chile, novelty in Norway.
- Awareness of food safety and official guidelines or recommendations was low.
- Findings highlight the need for clear consumption recommendations as seaweed becomes a more available food.

Macroalgae have been an important dietary component in many parts of the world for centuries, thus having a rich history as human food (1, 2). However, the practice and tradition of using macroalgae as food have changed over the years. While the culinary culture in Asia intensified the practice of eating macroalgae, it was drastically reduced in the Western diets, limited to artisanal practices and coastal communities at few scattered places (2–4). In certain parts of the globe, the consumption of macroalgae is associated with celebrations and feasts, while in other areas, it has been associated with times of economic hardship, scarcity, or even famine (5–9).

In recent years, there is a combination of factors enhancing the use of macroalgae as food. One important factor is represented by the popular trend toward a healthy food regime, linked to the view of macroalgae as healthy food or even as a ‘super food’ (10–13). This interest is fueled by attention on diverse bioactive components of macroalgae (e.g. alginate, carrageenan, carotenoids, fatty acids, fucoidan, phlorotannin, and phycobiliproteins), with known applications in the functional food and nutraceutical industries (4). In Western countries, there is an increase in lifestyles choices concerned with the environment, with special interest in healthy and sustainable products, vegetarian, or vegan diets (14). Another factor is represented by the intense globalization of products, previously inaccessible for many. Nowadays, macroalgae have entered the global food market, and a variety of wholefood macroalgae and macroalgae-containing products are now available for consumers (15, 16). Another factor is represented by the intense diversification of the gastronomic offer in restaurants, which is producing an substantial change in our diet (17–19). The consumption of macroalgae in Europe is inspired by Asian food with sushi maki, miso soup, and wakame salad products (20). An industry around macroalgae has emerged, ranging from high-end restaurants to entrepreneurs offering ‘harvest your own seaweed’-tours (21). Furthermore, regional macroalgae cuisines around the world have been rediscovered and reinvigorated, and many chefs have initiated a trend toward a new macroalgae gastronomy or ‘phycogastronomy’ (2).

Though still small in economic relevance when compared with terrestrial plants, the commercial interest in macroalgae has grown in the last years (22, 23). According the Food and Agriculture Organization (FAO) of the United Nations, global macroalgae production was approximately 35.8 million tons in 2019 (24), but the demand for macroalgae products by Western markets is expected to increase quickly in the future, due to an interest in alternative food sources (9). On the other hand, the inclusion of macroalgae in the diet may introduce some food safety issues (4, 16, 25). Chemical (pesticides, biotoxins, allergens, and some elements), microbiological, and physical hazards have been associated with the intake of macroalgae, although currently there are significant gaps in regulations concerning food safety hazards in seaweed (24, 26). Microbial contamination from *Salmonella*, *Bacillus*, pathogenic *Escherichia coli*, *Listeria*, *Staphylococcus aureus*, and *Vibrio* or fecal coliforms have been associated with macroalgae intake (27). Besides, macroalgae are known to be able to naturally concentrate minerals and metals that are several orders of magnitude higher than in the surrounding environment (28). And, for certain species of macroalgae, excessive intake of arsenic, cadmium, or iodine may be caused by their consumption (26).

In this tri-continental survey, across Asia, South America, and Europe, three distant countries, Chile, China, and Norway, were included. These countries all have exceptionally long coastlines where fisheries and aquaculture are well established and predominant economic activities, but they differ in cultural and culinary traditions. The three countries are among the top 10 countries in the production of macroalgae, with China leading with a volume of 20,122,041 tons in 2019 and Chile in the ninth place with 106,069 tons (24). In addition, Chile is the leading producer of wild harvested seaweed over the years, with 404,933 tons in 2019, while China and Norway are the second- and third-largest producers, respectively, contributing 16% (174,551 tons) and 15% (163,083 tons) of global production in 2019 (24).

Our objective was to compare the current use of macroalgae as food in a population sample from these three distant coastal countries. We investigated what kind of species and food items were consumed, as well as in what frequency these foods were consumed. Along with this, we explored the consumer’s perception of macroalgae as food.

## Materials and methods

### Participants and recruitment

Chilean, Chinese, and Norwegian adult students were invited to participate in the survey. Eligible participants were adults above 18 years of age who had been living in the respective countries for more than 10 years.

The survey was distributed via professional and personal networks, such as the student mailing list of Norwegian University of Life Sciences, the Chinese Academy of Sciences, or Universidad Andres Bello, social media (Facebook), and by snowball sampling (29) in the period from November 2021 to March 2022. Google Forms (https://docs.google.com/forms/) was used to administrate the questionnaires. A web-based survey was chosen due to the cost effectiveness and easy administration (19).

### Questionnaire

The questionnaire (Supplementary material 1) was divided into three sections (in addition to a section on demographic variables): 1) consumer knowledge of macroalgae as food; 2) food habits, preferences, and attitudes toward wholefood macroalgae and macroalgae-containing products; 3) a macroalgae-specific food frequency questionnaire (FFQ), using a recall period of 4 weeks.

The FFQ allowed us to specifically assess the intake of pure, raw, and whole food fresh and dry (and salted in the case of China) macroalgae and the use of macroalgae as an ingredient in different foods. The FFQ consisted of 32 items, divided into sections of fresh macroalgae, dried macroalgae, foods of dishes containing macroalgae, dietary supplements, and other food products containing macroalgae. All items had the following intake frequencies: Never/rarely; less than weekly; 1–3 times per week; 4–6 times per week; 1–2 times per day. Quantities of intake were not recorded. We included a checklist for 46 common types of macroalgae, which included common names according to each language, scientific names, and the common (English name). Respondents could also add other macroalgae products or species consumed during that period. The survey was available in four languages: Chinese, English, Norwegian, and Spanish.

### Ethical considerations and applications

To carry out the survey, we obtained the necessary permissions from Sikt, Norwegian Agency for Shared Services in Education and Research (previously known as Norwegian Centre for Research Data) (ref. no. 549243). Participation was voluntary, and participants were able to withdraw the participation consent at any time and without giving any reason. The information about the respondents was anonymized. All data were anonymized and stored at a safe server at the Norwegian University of Life Sciences.

### Statistical methods

The data collected were processed using descriptive statistics. SigmaPlot (version 14; Systat Software, Inc., San Jose, CA, USA) was used to plot the data and designate frequency distribution classes for further calculation of respective mean and standard deviations. Mean, standard deviation, and median values were calculated using Microsoft Excel 2010 Software.

## Results

A total of 585 adult respondents (155 in Chile, 295 in China, and 135 in Norway) answered the survey (Table 1), hereafter referred to as Chilean, Chinese, and Norwegian respondents. The average age was 27 years in Chile, 20 years in China, and 29 years in Norway, and across countries, more than half (60–73%) were women. Around half of the respondents (by country) live or have lived by the coast. More than half is or was studying at a university. In each country, more than two thirds of the total respondents did not follow any specific dietary pattern, and less than 10% followed one specific dietary pattern (Table 1).

**Table 1:** Socio-demographic description of the participants in the tricontinental macroalgae survey.

Category	Chile (*n* = 155)	China (*n* = 295)	Norway (*n* = 135)
**Gender**			
Woman	111 (72.0)^a^	177 (60.0)	98 (73.0)
Man	44 (28.0)	116 (39.0)	37 (27.0)
Other	0	2 (1.0)	0
**Age**	26.8 ± 9.5^b^	20.2 ± 2.8	28.5 ± 8.3
**Marital status**			
Single	125 (81.0)	287 (97.2)	50 (37.0)
Married/living with a partner	30 (19.0)	4 (1.4)	59 (44.0)
Other	0	4 (1.4)	26 (19.0)
**Level of education completed**			
No education	0	1 (1)	0
Lower Secondary School	0	0	0
Senior Secondary School	30 (20.0)	33 (11.0)	31 (23.0)
1–4 years university	78 (50.0)	202 (68.0)	53 (39.0)
More than 4 years university	47 (30.0)	59 (20.0)	51 (38.0)
**Location**			
By the coast	62 (40.0)	132 (45.0)	43 (32.0)
Not by the coast	72 (46.0)	147 (50.0)	57 (42.0)
Originally by the coast but not now	21 (14.0)	15 (5.0)	35 (26.0)
**Dietary patterns^c^**			
Pescetarian	4 (3.0)	20 (7.0)	13 (9.6)
Lacto-vegetarian	2 (1.0)	4 (1.0)	0
Ovo-vegetarian	5 (3.0)	9 (3.0)	0
Lacto-ovo-vegetarian	7 (5.0)	4 (1.0)	4 (3.0)
Vegan	0	4 (1.0)	8 (5.9)
Other	1 (0.5)	9 (3.0)	5 (3.7)
No specific dietary pattern	136 (88.0)	245 (83.0)	105 (77.8)

a The percentage of respondents are given in brackets.

b Mean ± SD.

c Pescetarian: Plant-based diet, including fish and seafood. Ovo-vegetarian: Plant-based diet, including eggs. Lacto-ovo-vegetarian: Plant-based diet including dairy products and eggs. Vegan: Purely plant-based diet.

### Consumer knowledge of macroalgae as food

In the three countries, over 90% of respondents stated that algae can be used as food (Table 2). Half of the Chilean and Norwegian respondents considered that macroalgae had positive health effects, while it is considered by 90% of Chinese respondents.

**Table 2:** General knowledge of the use of macroalgae as food of the participants in the tricontinental macroalgae survey.

Knowledge questions	Chile (*n* = 155)	China (*n* = 295)	Norway (*n* = 135)
**Can macroalgae be used as food?**			
Yes	140 (90.0)^a^	280 (95.0)	153 (98.5)
No	4 (3.0)	3 (1.0)	0
I don’t know	11 (7.0)	12 (4.0)	2 (1.5)
**Do you know an official recommendation regarding the amount of macroalgae you can consume?**			
Yes	2 (1.0)	4 (1.0)	13 (9.6)
No	134 (87.0)	291 (99.0)	17 (12.6)
I don’t know	19 (12.0)	0	105 (77.5)
**Do you believe macroalgae have positive health effects?**			
Yes	76 (49.0)	266 (90.0)	68 (50.4)
No	1 (1.0)	0	0
I don’t know	78 (50.0)	29 (10.0)	67 (49.6)
**Do you believe macroalgae contain many nutrients?**			
Yes	53 (34.0)	267 (91.0)	83 (61.5)
No	1 (1.0)	2 (1.0%)	1 (0.7)
I don’t know	101 (65.0)	26 (8.0)	51 (37.8)
**Do you believe macroalgae can contain toxic or negative substances for health?**			
Yes	46 (30.0)	241 (82.0)	56 (41.5)
No	6 (4.0)	10 (3.0)	3 (2.2)
I don’t know	103 (66.0)	44 (15.0)	76 (56.3)

a The percentage of respondents are given in brackets.

When asked about food safety issues, 30, 82, and 42% of the Chilean, Chinese, and Norwegian respondents, respectively, considered that macroalgae may contain toxic or harmful substances (Table 2). In Chile and China, 1% of the respondents stated that they know the existence of an official recommendation on the amount of macroalgae you can consume, while in Norway, this percentage reached 9% (Table 2). However, no respondents were able to give the content of such an official recommendation. More than 87% of the respondents from Chile and China answered that such a recommendation does not exist.

### Food habits, preferences, and attitudes toward pure macroalgae and macroalgae-containing products

**Fig. 1:**
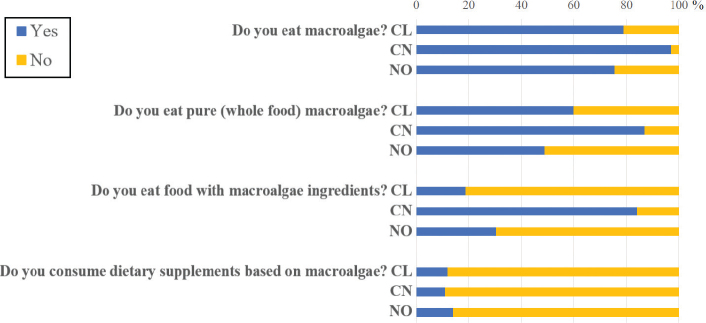
Percentage of use of macroalgae as food by the survey participants in the three countries (Chile: CL; China: CN; and Norway: NO).

Most of the Chinese respondents (97%) had macroalgae as part of their diet, while in the case of the Chilean and Norwegian respondents, around 75–79% included it in their diet (Fig. 1). Most Chinese respondents answered that they ate whole food (pure) macroalgae (87%), while in Chile and Norway, this percentage was much lower (60 and 49%, respectively). This difference among countries intensifies when considering foods containing macroalgae as ingredients; 84% of Chinese respondents answered that they consume such food, while only 20% of Chilean respondents and 30% of Norwegian respondents did (Fig. 1). A lower number of respondents from the three countries (11–14%) consume dietary supplements based on macroalgae.

**Fig. 2:**
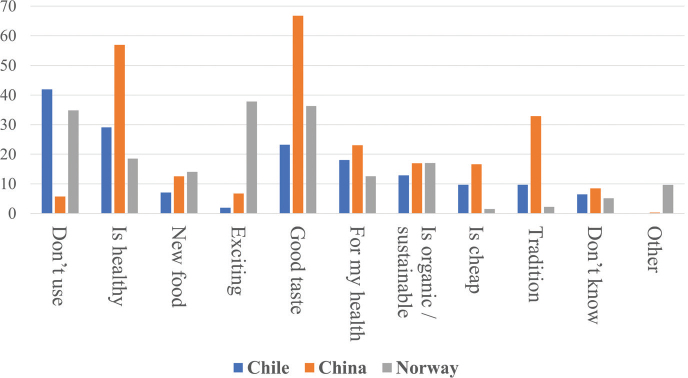
The main reasons why participants in this study used macroalgae as food. The values are expressed as percentage of the total of respondents for each country.

Consumers from the three countries answered that the main reason why they eat macroalgae products is because of the taste and because they were considered healthy foods. In addition, in China, the tradition of its use is very important, followed by Chile, while in Norway, the novelty of its use attracts the attention of consumers (Fig. 2).

The main source of information regarding the use of macroalgae as food in Chile was family and friends, followed by media (television, radio, and internet), and tradition. In China, the sources were media, followed by restaurants, tradition, schools, family, and friends. In Norway, the information sources were media and family and friends, followed by restaurants and schools (Supplementary material 2).

Chilean respondents obtained pure, whole food macroalgae mainly from markets, followed by commerce (common shops), and restaurants (Supplementary material 3A–B). In terms of products containing macroalgae as ingredients, Chilean respondents prefer to obtain it from common shops, followed by restaurants and wet markets. In China, the respondents’ main sources of whole food macroalgae products and food that contain algae as ingredients were commerce, followed by restaurants and wet markets. E-commerce was used by less than 30% respondents for these commodities. There was a small percentage that obtained macroalgae products from health food stores and others who harvest macroalgae themselves (Supplementary material 3A–B). In Norway, restaurants are the crucial place for the respondents to consume whole food macroalgae and food with ingredients containing algae, followed by commerce, health food stores, and e-commerce. Interestingly, 6–8% of the Norwegian respondents who eat macroalgae harvested their own macroalgae from the coast (Supplementary material 3A–B).

### Macroalgae-specific FFQ

#### Consumption of whole food macroalgae

**Fig. 3:**
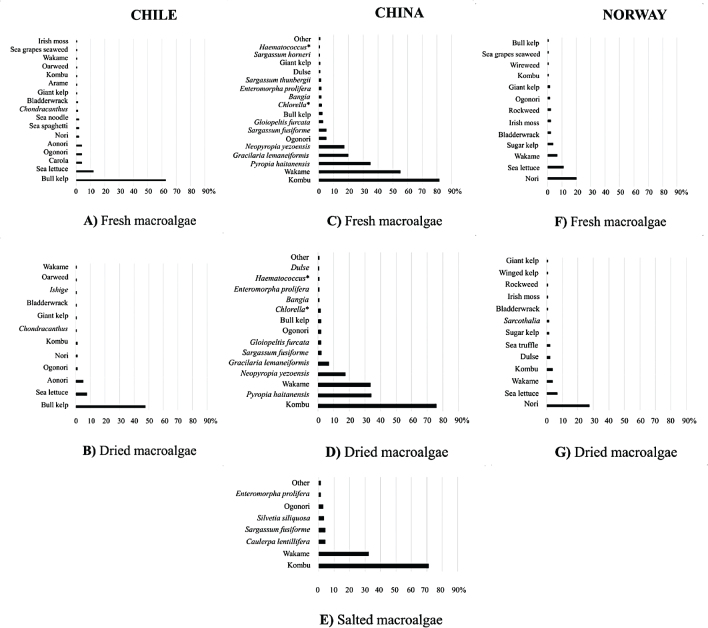
List of species names or as common product used by the questionnaire participants during the last four weeks prior to answering this study. Bars represent the percentage of pure, whole food macroalgae species (fresh, dry, or salted) consumed by respondents in each country. Chile: fresh (A) and dried (B); China: fresh (C), dried (D), and salted (E); and Norway: fresh (F) and dried macroalgae species (G).

The number of species of macroalgae used as whole food (fresh, dried, or salted) during the 4 weeks before answering the survey was very similar (between 17 and 19) among the three countries (Fig. 3).

From the FFQ, the large kelp species (brown macroalgae) were the dominant species consumed in Chile and China. *Durvillaea* spp. ‘bull kelp’ was the main preference by Chilean respondents, while in China, *Saccharina japonica* ‘kombu’ was the main preference; both were fresh, dried, or salted, followed by another kelp, *Undaria pinnatifida* ‘wakame’. The dominant species consumed in Norway belong to the red macroalgae, where ‘nori’ (probably including imported species of *Neopyropia*, *Porphyra*, and *Pyropia*) was the main preference (Fig. 3F–G). Red macroalgae were also popular in China (*Pyropia haitanensis*, *Gracilaria lemaneiformis*, and *Neopyropia yezoensis*), following the mentioned wakame in the order of preference of consumption (Fig. 3C–D). The green macroalga ‘sea lettuce’, which includes several species of the genus *Ulva*, was the second most consumed alga among Chilean and Norwegian respondents, but not the Chinese respondents.

### Frequency of consumption of whole food macroalgae

In terms of frequency of consumption, few respondents used whole food macroalgae daily. Three Chinese respondents consumed fresh and dried kombu and nori, and fresh ‘ogonori’ *Gracilaria* sp. One Chilean respondent used merely non-native bladderwrack *Fucus vesiculosus* (probably as a dietary supplement), and no macroalgae species was used with this frequency by Norwegian respondents.

A low percentage (<2%) of the total Chinese respondents used whole food macroalgae 4–6 times per week. They preferred both brown macroalgae (kombu and wakame) and red macroalgae (nori). Less than 1% of the total Chilean and Norwegian respondents consumed whole food macroalgae at this frequency, and they preferred green macroalgae (‘aonori’ and sea lettuce, respectively). At a frequency of consumption of 1–3 times a week, the percentage of Chinese respondents increased, especially using fresh kombu (18%) or nori (14%). At this frequency, Chilean consumers preferred fresh and dried bull kelp (<6%), while Norwegian consumers preferred sea lettuce (<4%, Supplementary material 4).

### Foods, dishes, and dietary supplements containing macroalgae as an ingredient

**Fig. 4:**
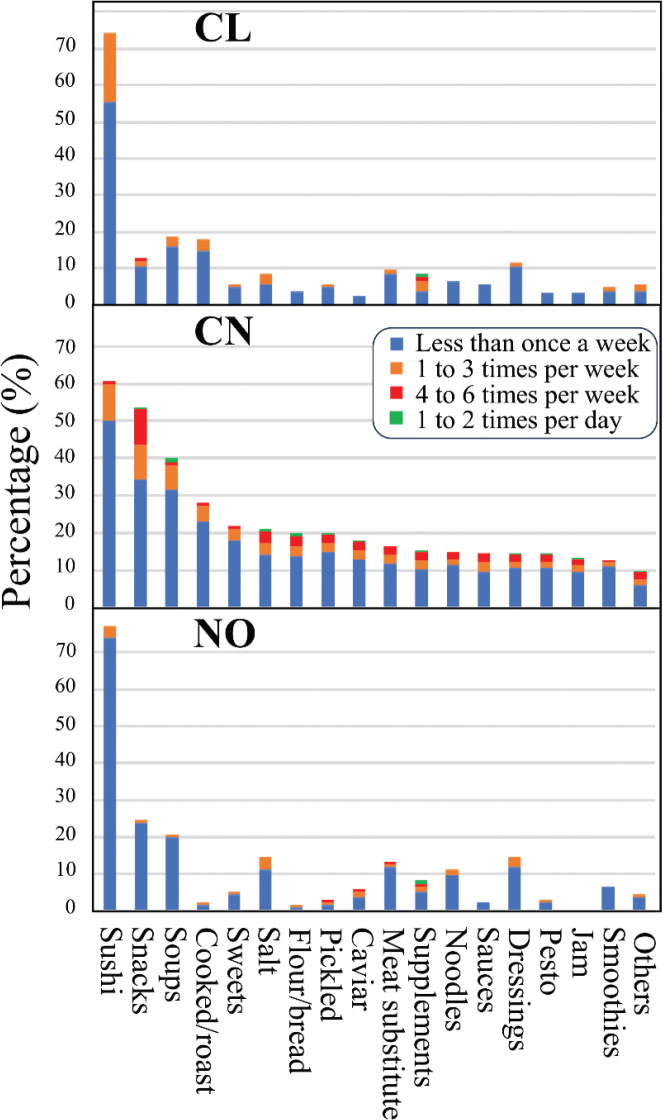
Percentage of participants (bars) classified by product consumed that contained macroalgae as an ingredient. The frequency of their consumption within the 4 weeks prior to answering the questionnaire is reflected by the different colors in the bars.

Figure 4 shows the intake frequency of foods and dishes using macroalgae as an ingredient for all three countries. The main preference in all three countries was sushi with nori, which was consumed by 74% of Chilean respondents, 61% of Chinese, and 77% of Norwegian respondents. This was followed by two products, snacks based on macroalgae and soups with macroalgae (13 and 54%, respectively, in Chile, 54 and 40% in China, and 24 and 21% in Norway). Chilean and Chinese respondents consumed cooked or roasted macroalgae (18% in Chile, 28% in China, and only 2% in Norway). Algae-based dressings and salt with macroalgae were also important in the three countries.

Each food preparations containing macroalgae as an ingredient included in our survey was consumed by at least 13% of total Chinese respondents. Sweets with macroalgae, flours and bread based on macroalgae, and pickled or fermented macroalgae were consumed by more than 20% of the respondents (Fig. 4B). In contrast, the consumption of most food products containing macroalgae in Chile and Norway did not exceed 13% of product preferences in the questionnaire (Fig. 4).

Only a small percentage of respondents (11–14%) in the three countries consumed dietary supplements based on algae (Supplementary material 5a). From the ones who did, tablets or capsules of algae were the preferred product (8–13%), followed by capsules of dietary fiber based on algae. Even though the numbers of users were limited, dietary supplements were one of the few products that were indicated to be used 1–2 times a day (Fig. 4). For more detailed information on type and source of dietary supplements with algae, see Supplementary material 5, Dietary supplements consumption.

## Discussion

### Use of macroalgae as food in the three countries

According to our results, the great majority of respondents of the three countries eat macroalgae (Fig. 1). China stands out from the other two countries as only 3% did not consume macroalgae, and there were no differences regarding the type of product consumed, whether pure macroalgae or as an ingredient. In both Chile and Norway, macroalgae were preferred as a separate food or whole food and much less as an ingredient in other food products.

In our study, Chinese respondents consumed the largest number of pure (whole food) macroalgae species (*n* = 19), of which kelp species (Asian kombu and wakame) were the main preference in terms of number of consumers and frequency of consumption in all categories of dried, fresh, or salted, followed by red macroalgae with different Asian nori species (Fig. 3, Supplementary material 4). This is in line with another study carried out in another Asian country by Zava and Zava (30), who found over 20 species of macroalgae included in meals in Japan. The current rising trend for the use of macroalgae in the Western world can be seen in our results, where consumers from Chile and Norway reported the use of 18 and 17 species during our study, respectively. Similarly, in another study from Norway, 96 different macroalgae products were collected from shops and Norwegian web shops, containing 18 different species of macroalgae (16). This is in line with our current study, showing a quite large variety of species consumed. In Norway, the red macroalgae ‘nori’ was preferred by the respondents, followed by sea lettuce in the current study. Nori is the most popular ‘species’ because probably sushi is a relatively common food in Norway. In Europe and America, the use of macroalgae as food has historically been in decline, where only certain localities (i.e. Brittany region of France) have maintained the use of some macroalgae as something persistent (2). A recent study in the Brittany region of France reported that respondents used around 10 macroalgae species as food in the last 12 months (although scientific names were not always given). The most frequent macroalgae species consumed were sea lettuce (26%), dulse (25%), wakame (including *Alaria esculenta*, 17%), nori (13%), sea spaghetti (*Himanthalia elongata*, 11%), sugar kelp (as royal kombu, 4%), kombu (including *Laminaria digitata* and *L. japonica*, 2%), and *Fucus* sp. (2%), which corresponded mostly to species present in France (20). In the UK, another study showed that *Ascophyllum nodosum* was the species most commonly found on the market (32%), followed by nori (14%), wakame (12%), kelp (11%), and dulse (15%), which also represent mostly preference for local products (15). In our study, China consumes more frequently species present in the country. Although, in Chile, respondents particularly prefer the use of one local species, the consumption of sushi is very high and frequent. While in Norway, the use of certain local species is observed, but on a very low scale, imported products are preferably used. In other similar recent studies from Sweden and Italy, the preferred macroalgae species were not given (see 19, 31).

### Knowledge and attitude toward macroalgae

In this study, we explored the knowledge and attitude toward the use of macroalgae as food in three different countries: Chile, China, and Norway. These are countries of vast coastlines, where fisheries, including macroalgae exploitation, are an established economic activity. Historically, there is an assumption that Asian countries include more macroalgae in their common diet (18). In our study, all three countries consumed a similar number of macroalgae species [*n* = 17–19] and a similar number of products containing macroalgae [*n* = 17–18]. Brown macroalgae were more frequently consumed in Chile and China, and in Norway, red macroalgae were most common. In Chile and China, tradition was an important reason for the consumption of macroalgae, while food novelty seemed to be a major factor in Norway. The main difference was observed in the frequency of consumption, where Chinese respondents used more species and products on a regular basis than Chilean or Norwegian consumers.

Only a few studies have focused on macroalgae from the perspective of consumer behavior (see 32). In our study, consumers of the three countries have a very clear perception that macroalgae can be used as food. Our results showed a general perception of algae as an appealing food due to its flavor and nutritional properties. Other studies have suggested a similar response in their respective countries such as Italy, Sweden, and the UK (15, 19, 21, 31). The respondents furthermore considered that macroalgae could have positive health effects.

### New and old trends in macroalgae consumption

Despite the increasing use of macroalgae as food, it is interesting to see how the country’s own tradition influences the preference for certain species. In the past, these foods were geographically segregated, while today, the import and availability in E-commerce have changed it radically (see Supplementary material 3). As mentioned, respondents stated that tradition was important in terms of consumption of macroalgae in Chile and China, while food novelty seemed to be a major factor in Norway (Fig. 2).

In countries where macroalgae were not a part of the culinary tradition, neophobia exerts a major influence on consumers’ acceptance of new food products (33). Nevertheless, the latter authors stated that the promotion of credence attributes of algae can be used to engage consumers potentially interested in new experiences. There are other cultural barriers that involve psychological variables, which can negatively affect the acceptance and use of macroalgae as food [reviewed by (34)]. These may be related to specific features of the product itself (strong flavor, dark color, and slimy consistence) or to sociological factors that are more difficult to identify (social status, social class, and ethnicity). This would explain why localities where macroalgae consumption persisted would tend to continue consuming the algae to which they had access over time (i.e. bull kelp in Chile or kombu and wakame in China), while localities who have not maintained this tradition are more open to imported products (as we saw in Norway). Similar results were seen in France (20) and in the UK (15). This does not mean that Norwegian consumers do not eat species available in their flora. Officially, the most common Norwegian species used for domestic consumption and food export are *Alaria esculenta*, *Saccharina latissima*, *Palmaria palmata*, and *Ulva* spp. (35). All these species were reportedly consumed by our Norwegian respondents. It is worth mentioning that nori and sea salad industries in Norway are still at an experimental level, and therefore, products available on the Norwegian market are mostly imported. According to our results, it is interesting to observe that the import of algal products is bidirectional (Asia to the rest of the world), but China also consumes imported macroalgae species, such as bull kelp.

The consumption of seaweed is on the rise in the Western world (36). The interest in macroalgae among consumers comes hand in hand with a greater diversification of the product offering, where traditional macroalgae-soups, stews, and salads are now accompanied by the consumption of macroalgae-based snacks, dressings, and jams. Although it is still possible to see that the frequency of the consumption of food products containing macroalgae as ingredients shows a great difference among Asia, Norway, and Chile, it is expected that this frequency will grow in the two latter countries once they become more widely available in the media and restaurants (see Supplementary material 2).

In the last 5 years, several local companies in Chile and Norway have started to develop products based on local macroalgae, including salt, chips, and condiments, usually with a legend related to native, sustainable, natural, and healthy. The mentioned study of Bouga & Combet (15) performed in the UK found that macroalgae were sold as whole or in sheets (23%) or were found in bread and confectionaries (19%), condiments (19%), sushi (14%), soup (7%), supplements (5%), snacks (4%), noodles and pasta (4%), and drinks (2%). The species that are mainly found in these foodstuffs were egg wrack (32%), dulse (15%), nori (14%), wakame (including *Alaria esculenta*, 12%), and kelp (*Laminaria longicruris*, 11%). The main origin of macroalgae was the UK (63%). In France, Ficheux et al. (20) found that the preferred food preparations containing macroalgae as ingredient included in their survey were sushi (71%), soups (48%), tartar and fish rillette (both 42%), salads (39%), salts (31%), and snacks and chips (24%), followed by drinks, cheese, bread, pasta, and mustard (all less than 18%). In the US, Laurel (37) made a study on consumer acceptability of bread containing macroalgae. In general, the author found a high level of willingness to eat seaweed among consumers, indicating that young generations of consumers are becoming more receptive to novel foods, and that this represents multiple opportunities for producers to use dried macroalgae for added umami flavor, nutrients, and novelty.

### Safety of macroalgae as food

Several food safety issues are associated with the consumption of macroalgae (16, 26, 27, 38). In 2023, European Food Safety Authority (EFSA) published an assessment of exposure of iodine and heavy metals through seaweeds in the European population (39). They found that exposure to cadmium, lead, and total arsenic was non-negligible among consumers, and that seaweed could be a cause of excessive iodine intakes, but this depends largely on the species consumed. In our study, awareness of potential food safety issues related to macroalgae was low in Chile and Norway, but in China, most of the consumers were aware of the potential risks and the presence of unwanted substances in macroalgae, especially regarding iodine and arsenic. Despite this, very few respondents in the three countries were aware of any official recommendations for macroalgae intake, and those who did mention an official regulation were unaware of the content of it. In Norway, the Norwegian food safety authorities have provided some advice to consumers in which they state that due to lack of knowledge, seaweed must generally be used with care and not eaten in large quantities (see 40). The determination of official recommendations for the consumption of macroalgae could be an important issue in the future, if macroalgae are moving from a niche food toward a more commonly consumed food. While brown macroalgae have very high levels of iodine, red macroalgae have a higher concentration of cadmium (27, 39). Nevertheless, high concentrations do not occur in all species by group of algae; therefore, more specific studies and recommendations could be beneficial. For now, the brown macroalgae destined for food are dominated by the order Laminariales and some Fucales (as in our results), but interest in other species of the order Ectocarpales has been increasing fast, for example, that is *Scytosiphon*, *Cladosiphon*, and *Petalonia* (41).

Furthermore, official recommendations must also consider the frequency of local consumption of macroalgae and associated products (39). In Japan, people consume on average 10.4 g of macroalgae per person per day (42). In South Korea and China, macroalgae consumption in adults is equal to 8.5 g a day and 5.2 g a day, respectively (43). In a recent study, the consumption of macroalgae by the French population was on average equal to 293 mg/day (20), which was much lower than the previously mentioned for Asian populations. Currently, European data on seaweed consumption are scarce, and more information on amounts, species, and frequency of intake is warranted (39). The amounts, species, and frequencies being consumed in Chile or in Norway need further investigations.

### Strengths and limitations of the study

We are not aware of any other study gathering information about the use of macroalgae as food from three of the major seaweed-producing countries. Some limitations of this study should be outlined. First, we sampled only a small number of participants using a convenient sampling method. We aimed at sampling through universities within limited geographical areas, and the external validity of our sample must be considered to be low. Furthermore, in Norway and Chile, we found a surprisingly high variety of macroalgae species consumed. We cannot rule out that the respondents in our survey had a particular interest in macroalgae as food. Second, no validated questionnaires existed on the use of macroalgae as food, and, thus, we developed our own questionnaires and a macroalgae-specific FFQ. Whether it has captured the true intake is uncertain. Furthermore, we notice that ‘western’ consumers are not always aware of what exactly they are eating in terms of macroalgae. Interestingly, color is a common source of error. For example, the common wakame salad, which is made of a brown macroalga, which turns green when being cooked, created confusion. Red algae like nori can turn into a brownish color when prepared in soups. Lack of knowledge of species may have introduced some error in our assessments. Third, we have not been able to assess any portion sizes of the macroalgae, and the intake in terms of amounts consumed is unknown. This would be an important topic for further research.

Even if this study has some major limitations, we believe our findings could be of interest, since intake and perceptions of macroalgae as food are understudied topics, especially in Norway and Chile. The results and experiences from this study could be used in future studies, assessing the intake of macroalgae at a more representative scale.

## Conclusion

Macroalgae have been subject to a renaissance as an appealing and healthy food, and the market related to macroalgae has been expanding rapidly. A number of species were found the diet either in pure form or as an ingredient in a variety of products in Norway, Chile, and China. However, as the use of algae in our diets intensifies, food regulations and clearly communicated dietary guidelines and food safety recommendations are required. This survey highlights the lack of awareness of potential food safety issues related to macroalgae among consumers. The findings indicate a need for increased public information and the development of guidelines for the safe consumption of macroalgae.

## Supplementary Material


