# Supplementary material for: Inclusion of macroalgae in the diet – a comparative survey from Norway, Chile and China
Source: Food Nutr Res. 2025 Jun 26;69:10.29219/fnr.v69.10856. doi: 10.29219/fnr.v69.10856 (PMC12255160; doi:10.29219/fnr.v69.10856)
Supplement: Supplementary file 1 [file FNR-69-10856-s1.docx]

**SUPPLEMENTARY MATERIALS**

**Supplementary material 1.** The English version of the questionnaire, including checklist for common types of macroalgae.


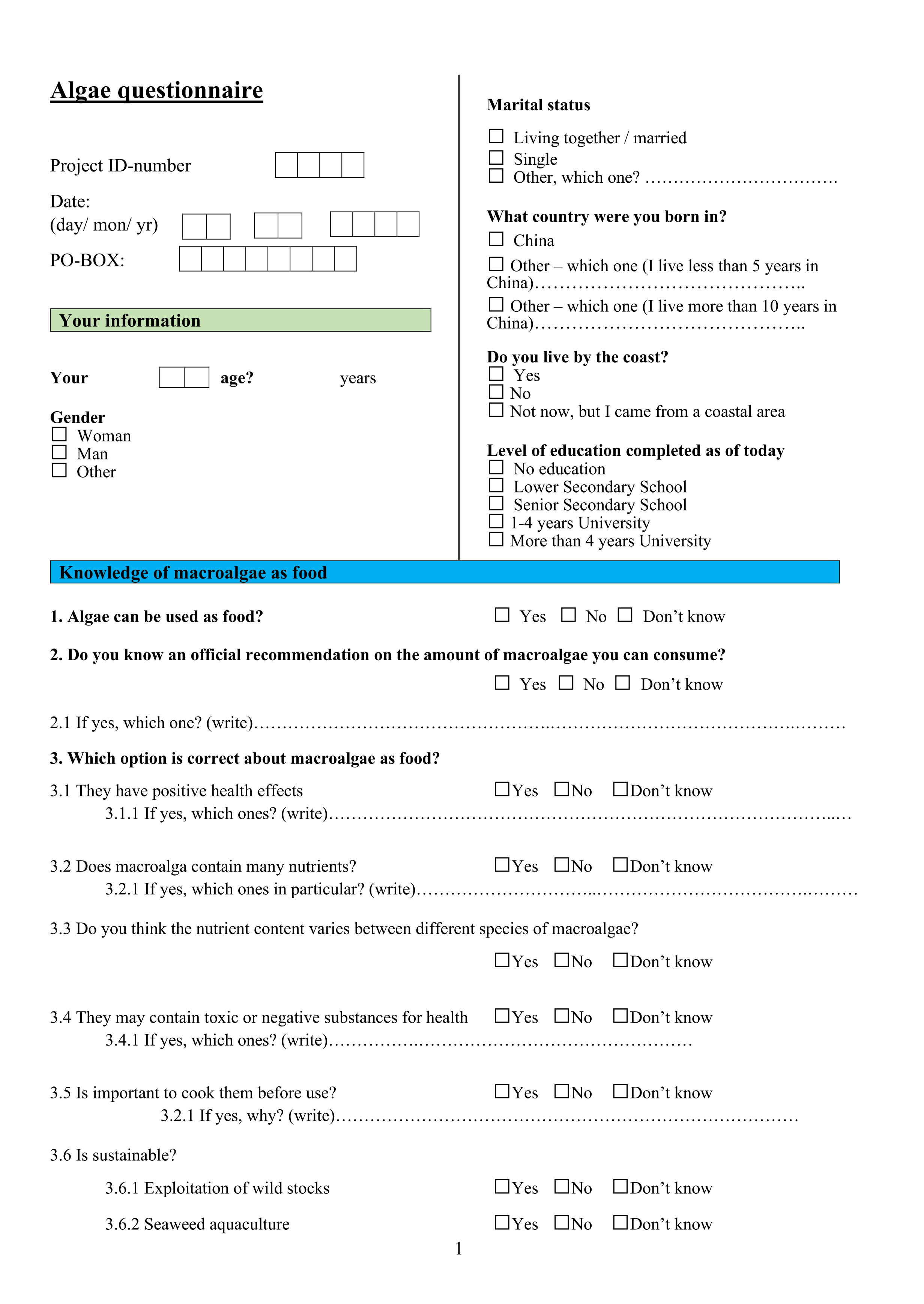


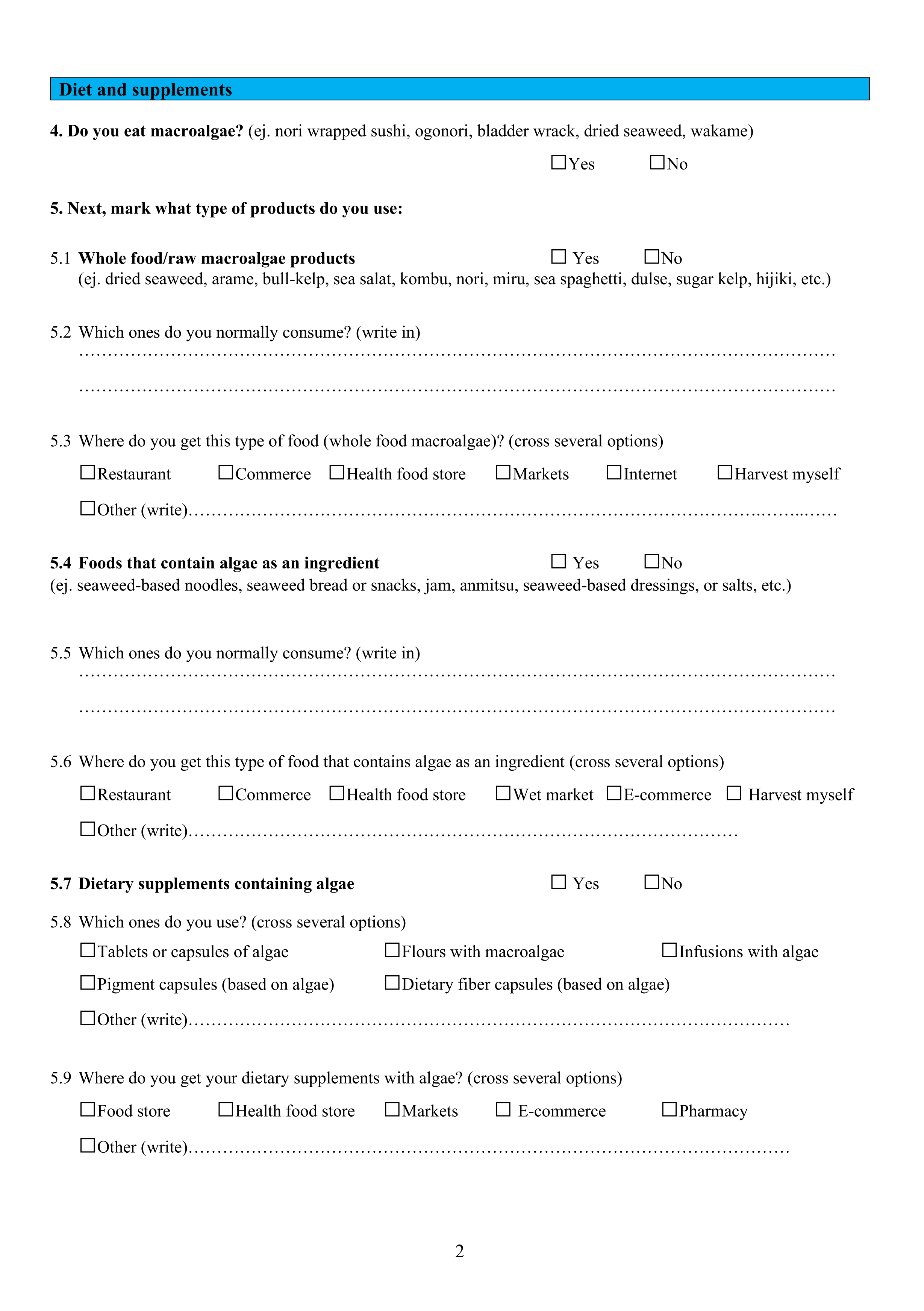

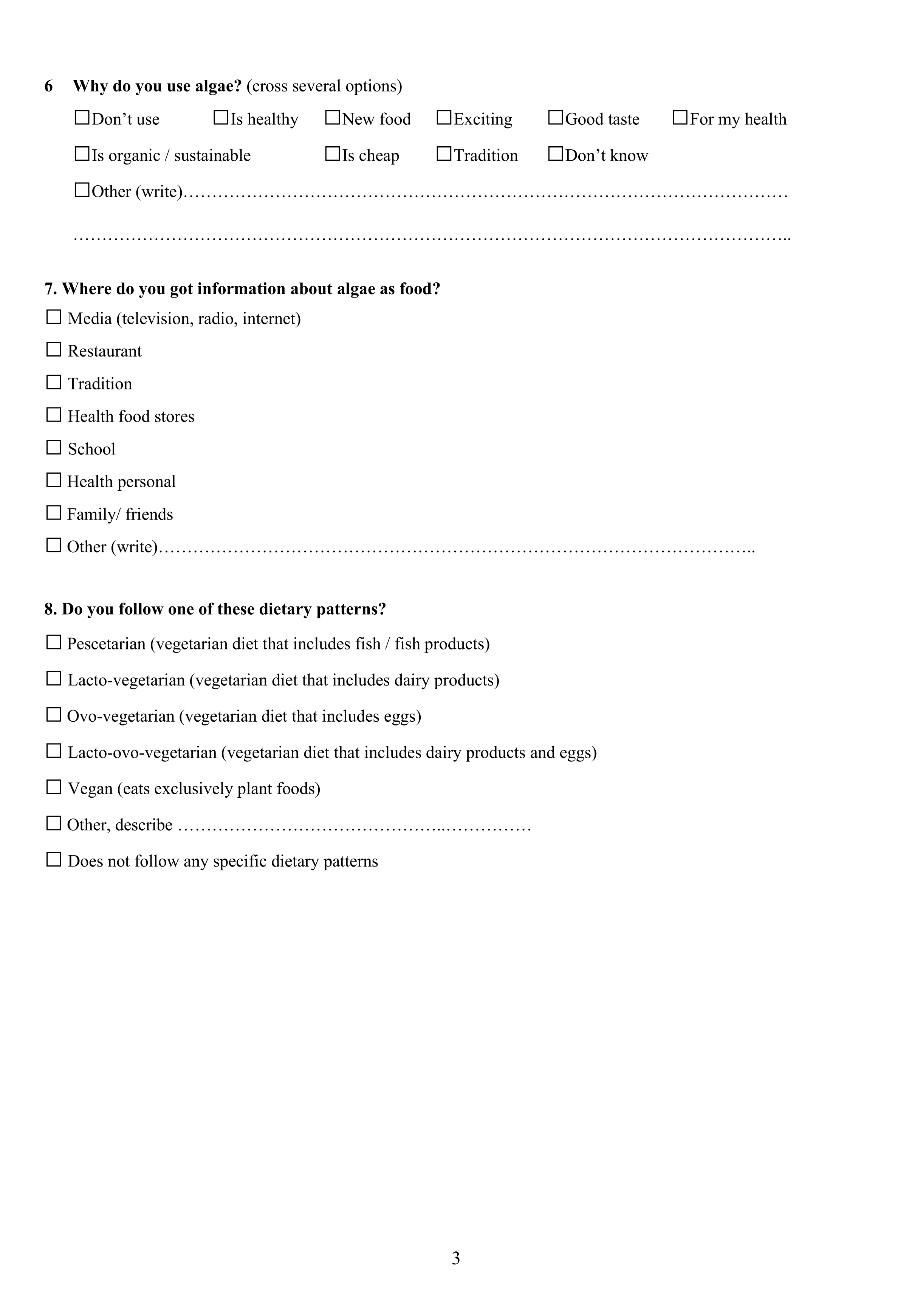

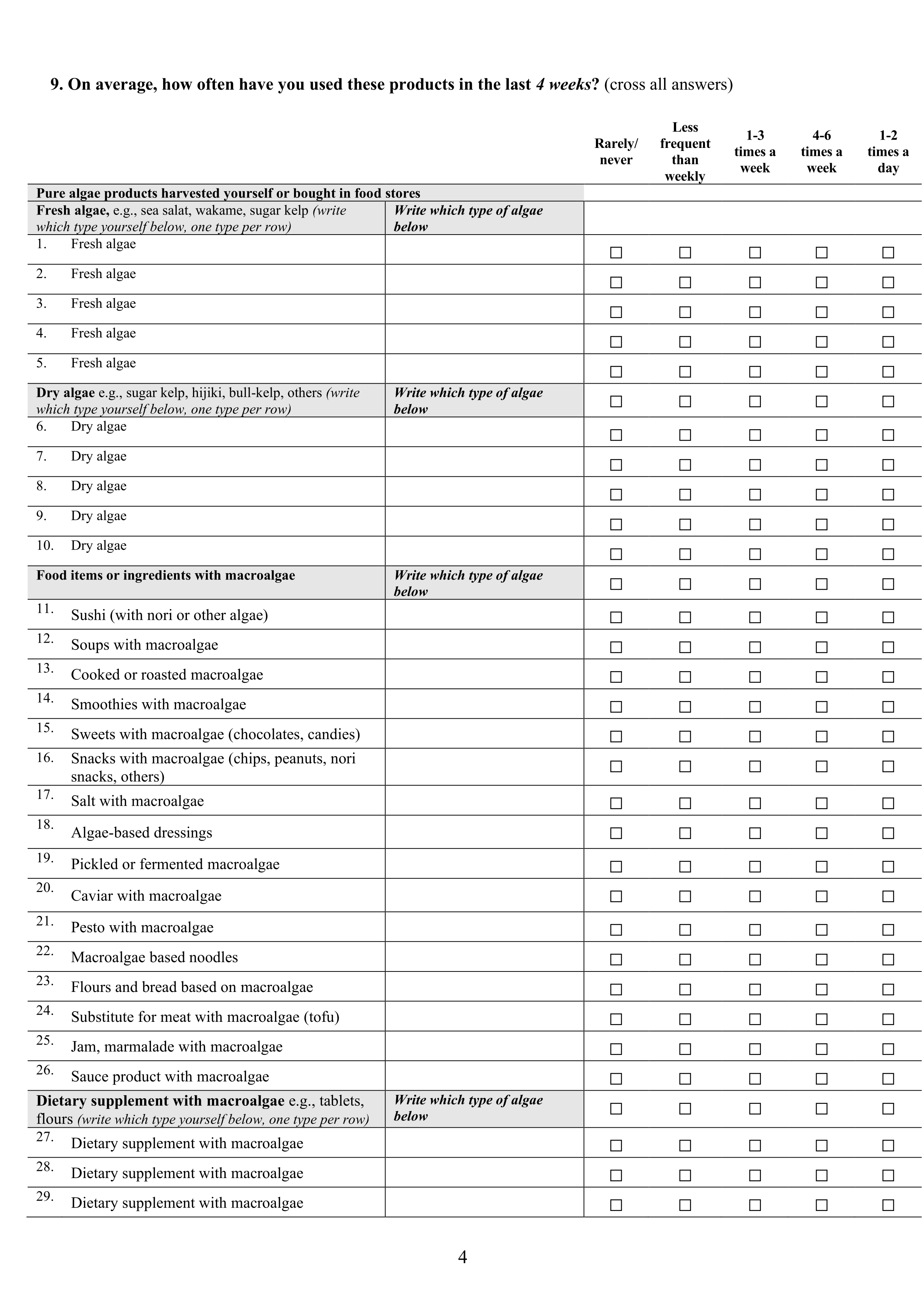

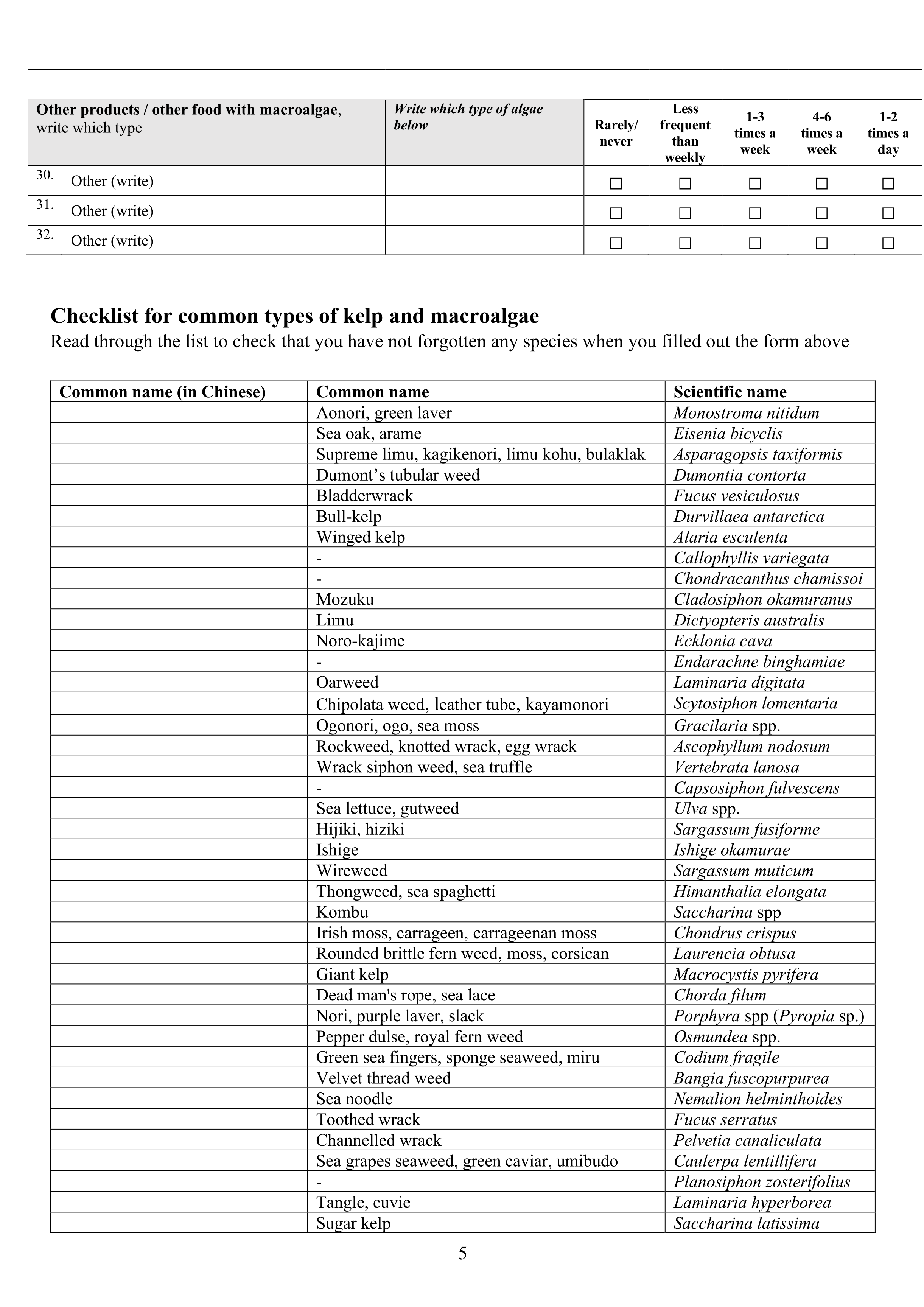

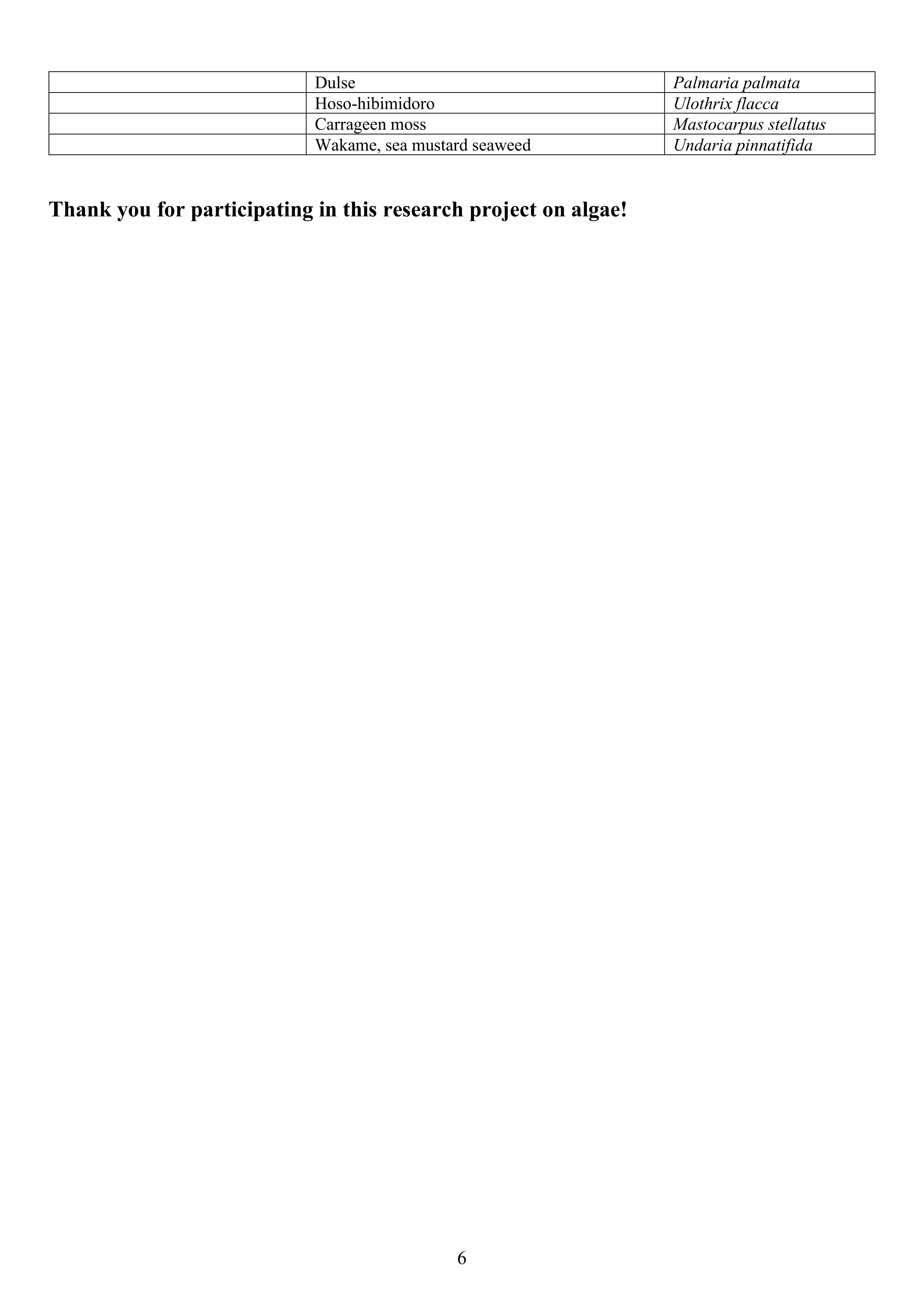


**
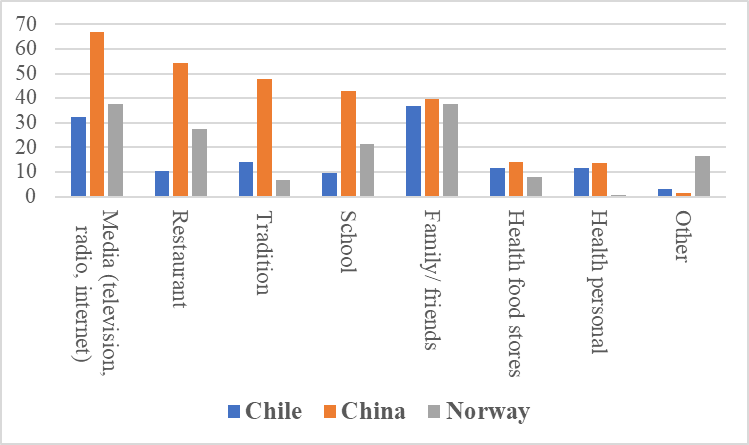
**

**Supplementary material 2.** Information source of algae as food for each country.

**a)
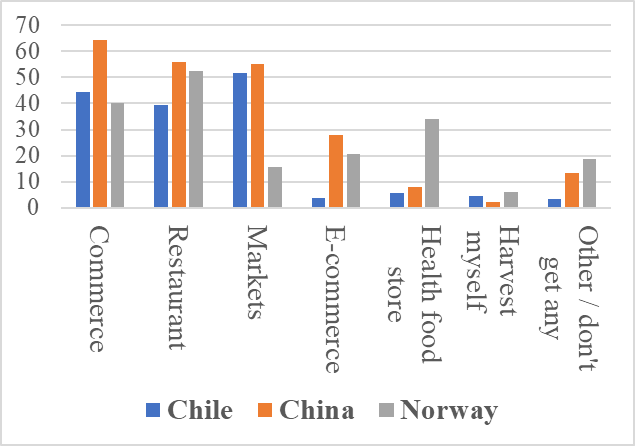
**

**b)
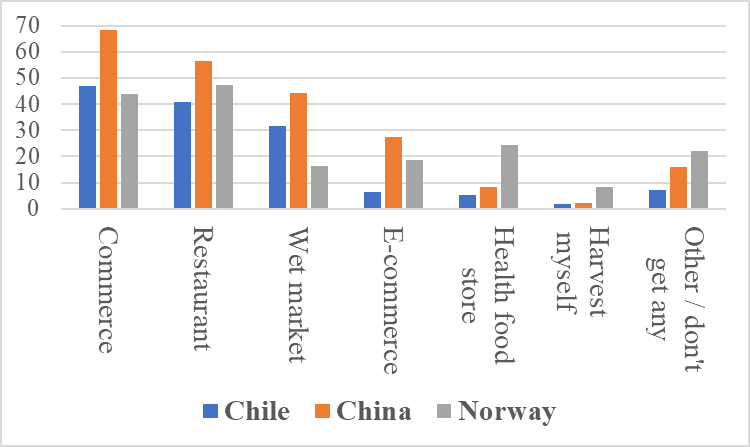
**

**Supplementary material 3.** Main source of whole food macroalgae and food that contain macroalgae as ingredient. **a)** pure macroalgae; **b)** products containing algae as ingredient for the three countries. Values expressed in percentage of the total respondents for each country.

**a)**
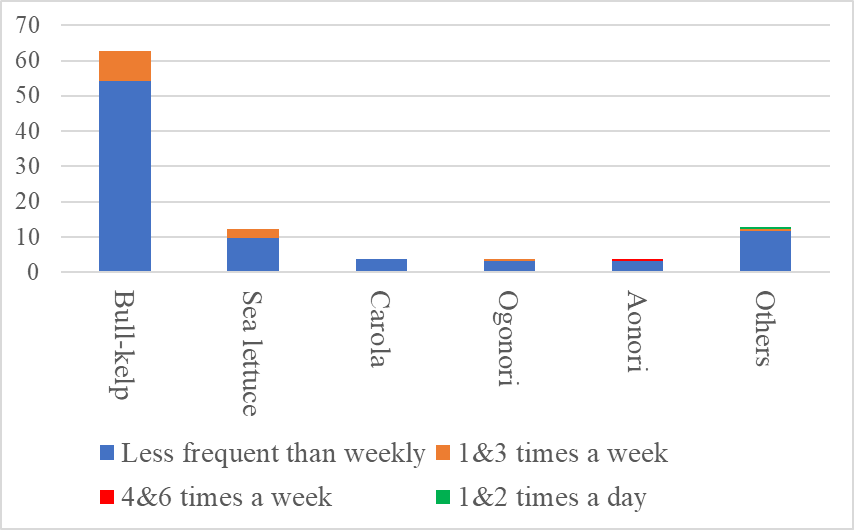


**b)**
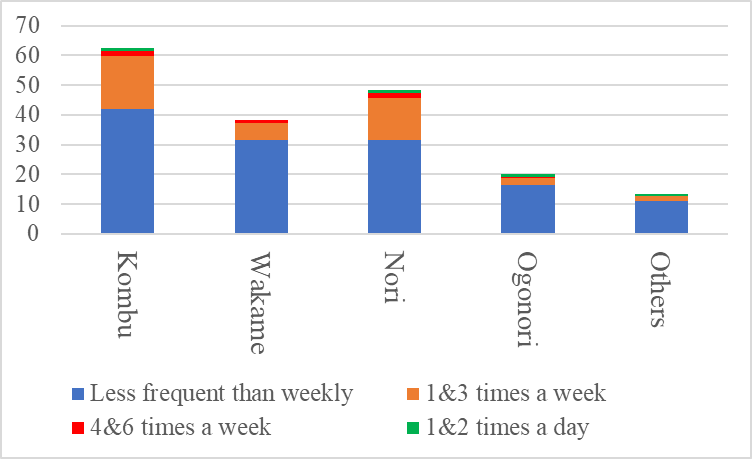


**c)
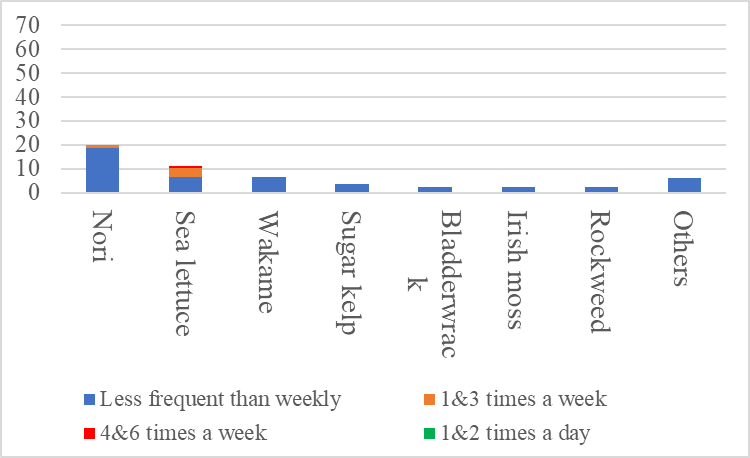
**

**d)
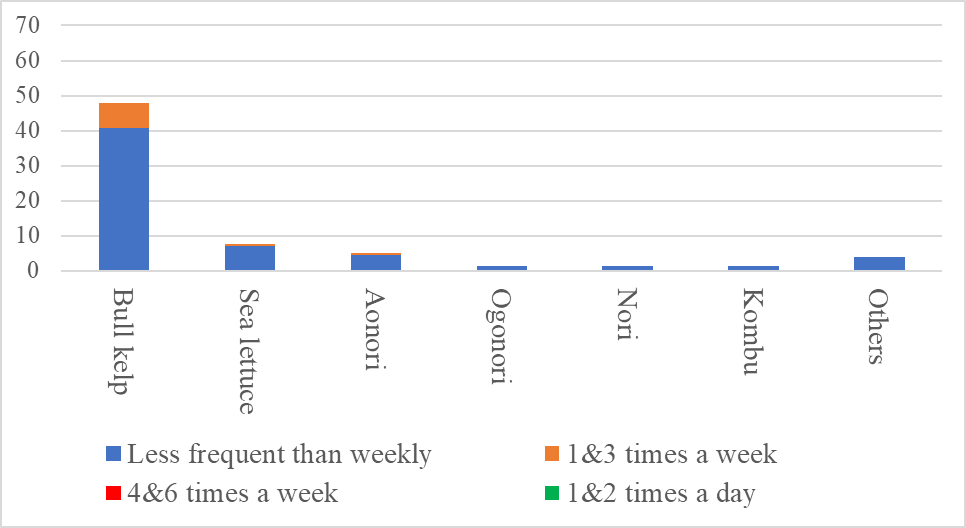
**

**e)
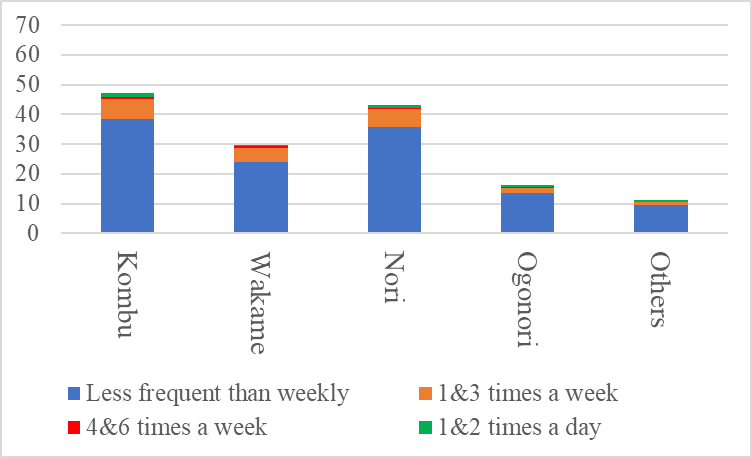
**

**f)
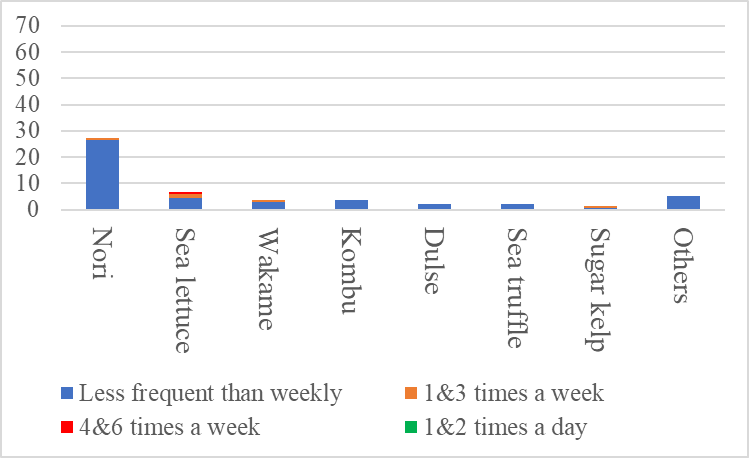
**

**Supplementary material 4.** Frequency of consumption of the main species of macroalgae eaten whole food (fresh or dried) as percentage of the total respondents of each country for the last four weeks prior answering the questionnaire.

**a)**

**b)**

**Supplementary material 5.** Dietary supplements consumption. **a)** Main used dietary supplements containing algae expressed in percentage of the total respondents for each country; **b)** main source of dietary supplements with algae.
